# Development and validation of language as an endpoint in Alzheimer’s Disease clinical trials

**DOI:** 10.1002/alz.087306

**Published:** 2025-01-03

**Authors:** Jessica Robin, Michael J Spilka, Mengdan Xu, William Simpson

**Affiliations:** ^1^ Winterlight Labs (Cambridge Cognition), Toronto, ON Canada; ^2^ Winterlight Labs, Toronto, ON Canada

## Abstract

**Background:**

Changes in the structure and use of language are well established clinical characteristics of Alzheimer’s disease. In recent years, there has been a concerted effort to objectively quantify these changes using the latest advances in Natural Language Processing (NLP) tools. Much academic research has been conducted to evaluate how these speech characteristics change with the course of illness, but they have yet to be elevated beyond exploratory endpoints in trials. Through a series of studies, we apply a validation framework with the goal of creating a speech based biomarker suitable for use in trials as a language endpoint.

**Method:**

In order to accurately capture the spectrum of disease, a biomarker should be able to 1) detect disease in case‐control samples, 2) be able to capture severity within clinical groups and 3) capture change over time. We conducted a series of studies leveraging multiple methods to assess how NLP derived speech and language features perform at each of these validation tasks.

**Result:**

Using machine learning based approaches, we were able to accurately differentiate patients with dementia from demographically matched controls with >80% accuracy. Numerous characteristics of speech, including syntactic complexity, information content, use of nouns and pronoun:noun ratios were shown to correlate with gold standard measures of cognition and function including the CDR, ADAS‐Cog, MMSE and ADCS‐ADL. Many of these same features also showed significant change over time, in line with traditional endpoints. Analytical strategies to combine these characteristics into composite scores have yielded measures which are correlated, but not identical to gold standard endpoints, with generally similar effect sizes and acceptable test‐retest reliability.

**Conclusion:**

Collectively, these studies indicate that speech and language can detect disease, align with severity and track clinical changes over time. While additional characteristics, including cross‐language performance, speech source optimization and precise evaluations of patient relevance are required, speech based digital biomarkers are a promising advance in the measurement of AD.

